# Prevalence of potentially traumatic events, depression, alcohol use, and social network supports among Chinese migrants: an epidemiological study in Guangzhou, China

**DOI:** 10.3402/ejpt.v5.26529

**Published:** 2014-12-09

**Authors:** Brian J. Hall, Wen Chen, Yan Wu, Fangjing Zhou, Carl Latkin

**Affiliations:** 1 Department of Psychology, University of Macau, Taipa, Macau (SAR), People's Republic of China; 2 Faculty of Medical Statistics and Epidemiology, School of Public Health, Sun Yat-sen University, Guangzhou, People's Republic of China; 3 School of Public Health, Sun Yat-sen Center for Migrant Health Policy, Guangzhou, People's Republic of China; 4 Department of Health Behavior and Society, Johns Hopkins Bloomberg School of Public Health, Baltimore, USA; 5 Department of Epidemiology, Johns Hopkins Bloomberg School of Public Health, Baltimore, MD, USA

**Keywords:** depression, alcohol abuse, social resources, migration, China

## Abstract

**Background:**

Addressing the health needs of Chinese migrants is a critical public health concern. Epidemiological studies are needed to establish the prevalence of potentially traumatic events (PTEs) and common mental disorders among Chinese migrants and identify protective community and social resources.

**Method:**

Utilizing random household sampling, we are in the process of recruiting a representative sample of Chinese adults (*N*=1,000) in two districts home to a large number of internal migrants. Data are collected using face-to-face interviews and participant self-report methods. Chinese versions of the Life Events Checklist, Alcohol Use Disorders Identification Test, Patient Health Questionnaire and the Social Support Rating Scale measured exposure to PTEs, alcohol use disorder, depression, and social support networks.

**Results:**

Preliminary results indicate a high proportion (68%) of the sample was exposed directly or indirectly to at least one PTE. The most commonly reported events were transportation accidents (43%), natural disasters (39%), and physical assault (26%). A total of 17% of the sample reported drinking consistent with having an alcohol use disorder. Moderate or severe depression was reported by 9% of the sample. The majority (75%) reported having three or more people to rely on for support, and 41% reported active participation in civic groups. Despite these strengths, only half the sample reported having trust in their community.

**Conclusion:**

Preliminary evidence from this population-level survey indicates high exposure to PTEs and a high potential burden of alcohol use disorders. The role of social networks will be explored as potentially useful for community-based intervention development.

